# Role of Endoplasmic Reticulum Stress in the Anticancer Activity of Natural Compounds

**DOI:** 10.3390/ijms20040961

**Published:** 2019-02-22

**Authors:** Patrizia Limonta, Roberta M. Moretti, Monica Marzagalli, Fabrizio Fontana, Michela Raimondi, Marina Montagnani Marelli

**Affiliations:** Department of Pharmacological and Biomolecular Sciences, Università degli Studi di Milano, 20133 Milano, Italy; patrizia.limonta@unimi.it (P.L.); roberta.moretti@unimi.it (R.M.M.); monica.marzagalli@gmail.com (M.M.); fabrizio.fontana@unimi.it (F.F.); michela.raimondi@unimi.it (M.R.)

**Keywords:** ER stress, natural compounds, cancer, apoptosis, unfolded protein response

## Abstract

Cancer represents a serious global health problem, and its incidence and mortality are rapidly growing worldwide. One of the main causes of the failure of an anticancer treatment is the development of drug resistance by cancer cells. Therefore, it is necessary to develop new drugs characterized by better pharmacological and toxicological profiles. Natural compounds can represent an optimal collection of bioactive molecules. Many natural compounds have been proven to possess anticancer effects in different types of tumors, but often the molecular mechanisms associated with their cytotoxicity are not completely understood. The endoplasmic reticulum (ER) is an organelle involved in multiple cellular processes. Alteration of ER homeostasis and its appropriate functioning originates a cascade of signaling events known as ER stress response or unfolded protein response (UPR). The UPR pathways involve three different sensors (protein kinase RNA(PKR)-like ER kinase (PERK), inositol requiring enzyme1α (IRE1) and activating transcription factor 6 (ATF6)) residing on the ER membranes. Although the main purpose of UPR is to restore this organelle’s homeostasis, a persistent UPR can trigger cell death pathways such as apoptosis. There is a growing body of evidence showing that ER stress may play a role in the cytotoxicity of many natural compounds. In this review we present an overview of different plant-derived natural compounds, such as curcumin, resveratrol, green tea polyphenols, tocotrienols, and garcinia derivates, that exert their anticancer activity via ER stress modulation in different human cancers.

## 1. Introduction

Endoplasmic reticulum (ER)is the organelle in eukaryotic cells, described for the first time by Porter et al. in 1945 [1] after analyzing chicken fibroblasts by using electron microscopy. It appears as a membrane network including elongated tubules and flattened discs that span a great area of the cytoplasm. ER consists of the smooth and the rough ER that can exist either as interconnected or spatially separated compartments [2]. It is physically in contact with mitochondria in specific regions called mitochondria-associated membranes (MAMs) that play a very important role in Ca^2+^ homeostasis [3].

The ER performs many essential functions including folding and post-translational processing of membrane-bound and secreted proteins, lipid synthesis, degradation of glycogen, detoxification, and Ca^2+^ storage and release [4]. The proper ER luminal calcium concentration is also essential for its protein folding and posttranslational modification actions since molecules such as chaperonins, protein disulfide isomerases (PDIs), N-glycosylating proteins, and other enzymes need the correct oxidoreductase potential to work appropriately [5].

Various stress factors, such as hypoxia, starvation, oxidative insults, changes in pH, Ca^2+^ depletion, hypoglycemia, ATP depletion, and viral infections, can disturb ER homeostasis. All these aspects can interfere with correct protein folding, finally leading to the accumulation of misfolded or unfolded proteins, generating a condition known as ER stress [6]. In response to such a stress state, cells have evolved an evolutionary conserved signal transduction pathway called UPR (unfolded protein response) whose principal aim is to restore ER homeostasis [7]. Three different ER transmembrane sensors, inositol requiring enzyme1α (IRE1α), protein kinase RNA(PKR)-like ER kinase (PERK), and activating transcription factor 6 (ATF6) detect the unfolded or misfolded proteins accumulated in the ER and initiate three distinct UPR branches, respectively, to overcome stress and restore homeostasis. However, when the stress conditions are too intense and cannot be turned back, the UPR activates a cell death pathway, generally via intrinsic apoptosis involving the mitochondria [6]. For this reason, under toxic and unresolved stress conditions, UPR transforms the cell destiny from survival to death [8]. However, novel ER stress-independent functions of UPR are here described; in macrophages X-box binding protein1 (XBP1) mediates the secretion of pro-inflammatory cytokines by the Toll-like receptor, independently of ER stress activation [9]. XBP1 is important for secretory cells and for the formation of plasma cells. In B cells that differentiate to plasma cells, XBP1 activation is differentiation-dependent and not a UPR-dependent event [10].

Cancer represents one of the leading causes of death worldwide. The analysis presented in the GLOBOCAN report estimated 18.1 million new cases of cancers and 9.6 million cancer deaths in the world in 2018 [11].

Over the past several decades, the discovery of plant-based drugs contributed to develop anticancer drugs, some of which have been historically approved in United States by the Food and Drug Administration (FDA) such as paclitaxel, camptothecin, vincristine, and their analogs [12]. Natural compounds represent a heterogeneous ensemble of structures that can be exploited to develop future effective drugs. Natural compounds could be originated from plants, animals, and produced from microbes, and many of them have shown a low risk of side effects in clinical trials [13]. Natural compounds can be utilized in different manners in cancer management: as chemopreventive [14], as chemotherapeutic [15], or chemosensitizer agents [16]. For this reason, the research for new bioactive molecules present in natural compounds with antitumor capacity is highly encouraged.

In this review, we will examine some classes of natural compounds showing the ability to induce ER stress-related death in cancer cells. In particular, we will focus on preclinical studies (in vitro and in vivo approaches) by reporting the ER stress-related anticancer activity exerted by natural compounds, such as curcumin, resveratrol, green tea polyphenols, quercetin, garcinia, tocotrienols, and new recent other compounds, in different types of human cancers.

## 2. Endoplasmic Reticulum Stress Response Pathways

### 2.1. UPR

The UPR represents a cellular stress response starting in the ER, controlled by three distinct sensors, IRE1α, PERK, and ATF6. Normally the ER resident chaperone, known as glucose-regulated protein 78 (GRP78) or binding immunoglobulin protein (BiP) is bound to the ER luminal domain of the three sensors, maintaining them in an inactive state [17]. Once detached from BiP, IRE1 and PERK form homodimers or oligomers, and through autophosphorylation, activate their downstream pathways [17].

Upon release by BiP, the active form of ATF6 moves to the nucleus and promotes the transcription of different ER chaperones [8].

### 2.2. IRE1 Pathway

The IRE1 pathway is the most evolutionarily conserved arm of the UPR, activated in different processes, both physiological and pathological such as those inducing ERAD (ER-associated degradation) [18], lipid synthesis [19], and protein secretion [20]. IRE1 is a type I protein receptor with an N-terminal ER luminal sensing domain and a cytosolic C-terminus domain enclosing both an endoribonuclease domain and a Ser/Thr kinase domain. In humans, there are two IRE isoforms, IRE1α and IRE1β; the first one is expressed ubiquitously on the ER membranes, while the second one is found only on the epithelial cells of the gastrointestinal tract [8]. Upon ER stress condition, IRE1α dissociates from BiP, dimerizes, and autophosphorylates, converting to its active form. Once active, IRE1α triggers its endonuclease activity responsible for X-box binding protein1 (XBP1) mRNA splicing. XBP1s (XBP1 spliced) mRNA encodes for a stable transcription factor targeting a range of genes involved in pro-survival responses [21]. However, IRE1α, in its active state, can activate another downstream signal through post-transcriptional modification of different substrates via regulated IRE1-dependent decay (RIDD) whose activation tends to trigger apoptosis. Hence, IRE1α via oligomerization induces XBP1 mRNA splicing, whereas dimerization induces RIDD. Active IRE1α also interacts with tumor necrosis factor receptor-associated factor 2 (TRAF2) leading to the increment of apoptosis signal-regulating kinase 1 (ASK1) and JUN N-terminal kinase (JNK), and in turn, induces apoptosis [22].

### 2.3. PERK Pathway

PERK is an ER transmembrane protein associated with BiP in its inactive form, but upon dissociation from BiP in response to UPR triggering, it is activated by oligomerization and autophosphorylation [8]. Active PERK attenuates mRNA translation and prevents the arrival of new proteins into the ER compartment. This action is mediated by phosphorylation-mediated inactivation of the eukaryotic translation initiation factor 2 (eIF2α). The phosphorylation of eIF2α blocks the recycling of eIF2α in its active GTP-bound state, needed for starting polypeptide chain synthesis, leading to the attenuation of general protein translation. This process is crucial for decreasing the ER protein burden and to resolve ER stress [23]. This block in protein translation is not absolute; indeed, in the meantime, eIF2α phosphorylation paradoxically regulates the expression of activating transcription factor 4 (ATF4), a member of the CCAAT/enhancer binding protein family (C/EBP) family of transcription factors [24]. ATF4, in turn, regulates the expression of genes involved in the restoration of normal cellular homeostasis. Among its target genes, there is the pro-apoptotic C/EBP homologous protein (CHOP) [25]. CHOP and ATF4 upregulate the transcription of growth arrest and DNA-damage-inducible protein 34 (GADD34), which in turn causes the dephosphorylation of eIF2α. If ER stress is irreversible, ATF4-CHOP activation can induce the apoptotic pathway [26].

### 2.4. ATF6 Pathway

ATF6 is an ER transmembrane protein and a member of the leucine zipper family of transcription factors [27]. Two ATF6 homologues are expressed in mammalian cells: ATF6α and ATF6β. Following UPR activation, ATF6α moves to the Golgi apparatus where it is processed by site-1 and site-2 proteases (S1P and S2P) and is transformed in a cytosolic fragment: cleaved ATF6α. In this active form, it translocates to the nucleus and acts as a transcription factor regulating the expression of genes presenting ATF/cAMP response elements or ER stress response elements (ERSE) within their promoter, such as BiP, and protein disulfide isomerase (PDI). Unlike ATF6α, ATF6β is not crucial in responses to UPR or regulation of ER chaperones. ATF6β has been shown to inhibit ATF6α-mediated activity during UPR [28]. A study also shows that ATF6α regulates the transcription of ERAD components. Levels of ERAD components, including ER degradation-enhancing α-mannosidase-like protein (EDEM), hydroxymethyl glutaryl-coenzyme A reductase degradation protein 1 (HRD1), and Herp, were found to be lower in ER stress-induced ATF6α^−/−^ mouse embryonic fibroblasts (MEFs) compared to ATF6α^+/+^ MEFs. While ATF6 activity is mainly pro-survival, during severe and sustained ER stress, it can increase CHOP expression that is associated with cell death [29].

## 3. ER Stress Mediates UPR for Anticancer Strategies

ER stress is primarily a pro-survival adaptive response against different types of cellular insults.

However, in the presence of severe, prolonged, and sustained ER stress conditions, ER stress-mediated UPR might fail to re-establish ER homeostasis and switches from pro-survival to pro-death mechanisms [6]. Although the molecular mechanisms related to this switching are not completely understood, it has been described that UPR utilizes some of the sensors and executioners of pro-survival components to activate a pro-death pathway in response to severe ER stress [8]. Cancer cells adapt UPR to alleviate the ER stress condition as a survival approach for progression [30]. UPR in cancer has also been described to participate in mechanisms involved in resistance to chemotherapy or radiation [31]. Some studies demonstrated that prolonged and severe ER stress can induce apoptosis, offering an interesting therapeutic rationale for suppression of cancer through the accumulation of unfolded proteins [8].

### 3.1. Pro-Apoptotic Signals Involving IRE1α-XBP1

The IRE1 sensor is usually associated with pro-survival effects during ER stress conditions through the induction of different chaperones [32]. During severe and sustained ER stress, it has been shown that IRE1α can activate a pro-apoptotic signaling. Activation of JNK represents one of the better-known mechanisms observed in the pro-death activity of IRE1α. This ER stress sensor activates JNK and IRE1α knockout to reduce JNK activity, suggesting that IRE1α represents an upstream activator of the JNK pathway. TRAF2 can mediate the JNK activation by IRE1α [33]. In turn, JNK induces apoptosis by increasing reactive oxigene species (ROS) production, by enhancing the expression of pro-apoptotic BH3 only members and, in the opposite way, by reducing the expression of anti-apoptotic Bcl-2 family members. The pro-apoptotic proteins Bcl-2-associated X protein (Bax) and Bcl-2 homologous antagonist/killer (Bak) form complexes with cytosolic region of IRE1α in response to ER stress inducers and are essential for IRE1α signaling [34]. IRE1α, as previously discussed, could regulate XBP1 mRNA splicing to produce mature XBP1. Different studies indicated that the IRE1α-XBP1 signaling was observed in different human cancers including breast and hepatocellular cancer [35]. In breast cancer cells, XBP1’s active form increases the tolerance of cells to hypoxia [20].

Under sustained ER stress, IRE1α stimulates activation of RIDD to promote apoptosis. The molecular mechanisms of RIDD-regulated apoptosis are still unclear but recently it has been proposed that RIDD inactivates anticaspase-2 pre-miRNAs, whose cleavage leads to the generation of active caspase-2, which has a pro-apoptotic role [18].

### 3.2. Pro-Apoptotic Signals Involving PERK-eIF2a-ATF4/CHOP

Upon severe stress conditions, active PERK phosphorylates eIF2α, which in turn activates ATF4. The transcription factor ATF4 binds the promoter region of the *CHOP* gene, increasing its mRNA expression and subsequently its protein levels [21]. CHOP represents a crucial player in ER stress-mediated cell death and all three branches of UPR can affect CHOP expression [36]. During persisting ER stress, ATF4 and CHOP promote cell death by activating genes involved in protein synthesis, such as GADD34 and ERO1α (endoplasmic reticulum oxireductin1α) [37]; GADD34, whose upregulation represents a pro-apoptotic mechanism depending on CHOP expression, induces the dephosphorylation of eIF2α and thus restores protein synthesis, whereas ERO1α, which is involved in the oxidation of PDI, leads to a condition of hyper-oxidation in ER [30]. By augmenting ERO1α expression, CHOP also promotes Ca^2+^ release via channel inositol 1,4,5-triphosphate receptor (IP3R) from ER to the cytoplasm. The increase of Ca^2+^ in the cytoplasm activates the calcium/calmodulin-dependent protein kinase II (CaMKII), which acts as an upstream molecule regulating apoptosis [38].

CHOP can definitely activate a death program inducing both extrinsic and intrinsic apoptotic pathways. CHOP up-regulates death receptor 5 (DR5) together with caspase-8 activation, which in turn generates the truncated form of Bid (tBid) and transports it into the mitochondria [39]. On the other hand, CHOP can also trigger the intrinsic apoptotic pathway, decreasing the expression of anti–apoptotic Bcl-2 and Bcl-xL proteins, while increasing the expression of pro-apoptotic proteins such as Bak, Bax, Bim, Puma, and Noxa [40]. Besides the interplay between ER and mitochondrial intrinsic apoptosis pathway, activation of ER-resident caspase, during ER stress, represents another mechanism to induce apoptosis. Indeed, under ER stress, the active form of rodent caspase-12 and human caspase-4 activates caspase-9, which in turn activates caspase-3, triggering apoptosis [41].

### 3.3. Pro-Apoptotic Signals Involving ER Ca^2+^ Release

The perturbation of Ca^2+^ levels represents another method regulating the intrinsic apoptosis pathway involving ER. ER-associated caspase-8 cleaves BAP31, an integral ER membrane protein forming the p20 fragment, thus abolishing its pro-survival function [42]. Moreover, the p20 fragment exerts pro-apoptotic signals by releasing Ca^2+^ from ER into the cytosol. Once in the cytosol, Ca^2+^ is subsequently internalized by the mitochondria, resulting in mitochondrial fission and cytochrome *c* release. Edelfosine, an antitumor agent, induces the cleavage of BAP31 with the formation of pro-apoptotic p20 fragment and causes a gradual Ca^2+^ release from ER in HeLa cells [43].

### 3.4. The Role of UPR in Cancer Cells

Numerous studies reported that UPR is often upregulated in cancer, suggesting its supportive role to tumor progression [31]. Indeed, ER stress and UPR are involved in all different stages of tumor progression. In the early stages of transformation, the high demand for proteins to sustain growth induces ER stress that in turn activates a pro-survival UPR, increasing the protein folding capacity. For example, the inhibition of IRE1α RNAse activity decreases breast cancer cell growth in vitro [44]. During tumor progression, extrinsic stress factors for tumors, such as hypoxia, nutrient starvation, and high cell density, induce ER stress, and the resulting adaptative UPR can promote the expression of pro-angiogenic factors to resolve hypoxia and can rewire the metabolic pathways to increase nutrient supply. IRE-XBP1’s signaling can sustain cancer growth in hypoxic condition, likely through interaction with HIF1α [45]. In the metastatic stage, the epithelial to mesenchimal transition (EMT) allows for the loss of cell to cell contacts, favoring the formation of a migratory and invasive phenotype. Activation of ER stress has been described to help cancer cells in EMT by overcoming the stress of cell detachment and this involves both the IRE-XBP1s and PERK-eIF2α-ATF4 signaling pathways [46,47]. Finally, during chemotherapy, UPR is an important mechanism that can induce chemoresistance in cancer by enhancing drug efflux from the cell. It has been reported that knockdown of BiP, ATF6, ATF4, and XBP1s can resensitize cancer cells to chemotherapy [48,49]. Constitutive activation of cytoprotective UPR signaling supports cancer cell progression and chemoresistance. However, upon prolonged or severe ER stress condition, a persistent UPR can induce a pro-death program. Therefore, to obtain an antitumor activity through the ER stress modulation, two approaches can be followed: on the one hand, the adaptative UPR can be inhibited for rendering the cells intolerant to ER stress and on the other hand, a sustained ER stress, activating pro-death signaling, can be induced.

## 4. Natural Compounds

For millennia, plants represented fundamental components of human life. We use plants not only as a source for food, beverages, cosmetics, and dyes, but also as drugs in medicine. A healthy diet including the daily consumption of fruits, vegetables, and spices can be an effective way to prevent the development of cancer, on the basis of the bioactive compounds contained in these foods [50]. These molecules are also known as phytochemicals, or nutraceuticals, and can represent plant secondary metabolites. The use of natural products constitutes a promising intervention to prevent, inhibit, or reverse the process of carcinogenesis [51]. Natural compounds are characterized by antioxidant, anticarcinogenic, antimutagenic, and detoxification properties [52] that could be used to produce new drugs with effective anticancer activity.

Although many antitumor compounds have been demonstrated, unfavorable side effects and drug resistance still represent limitations of current anticancer therapy [53]. Indeed, the side effects of traditional drugs prevent clinical outcomes. Thus, researchers need to identify new agents to develop more reliable therapies. Once the curative potential of plant-derived drugs is known, it is possible to investigate the synergistic or additive effects that result from the assortment of compounds occurring in plants [54]. In addition, the toxicity of natural compounds is usually less compared to traditional drugs. Thus, their use is supposed to expand the efficacy of traditional anticancer agents, and at the same time, to decrease their toxicity. Natural compounds and traditional agents, combined together, would potentially lower the dose of the classic drugs that is needed to obtain the therapeutic outcome, consequently limiting their detrimental effects [55]. To reach a successful anticancer treatment strategy, it is important to better clarify how the natural compounds interact with cellular targets. Hence, affecting the increase of ER stress related protein response could represent an interesting approach to modify the homeostasis of the ER in cancer cells in order to activate apoptosis. Although ER stress can promote the survival of cancer cells, under specific conditions, it can support cell death. Different natural compounds have been reported to induce ER stress-related apoptosis in malignant cells [30,56,57].

**Figure 1:**
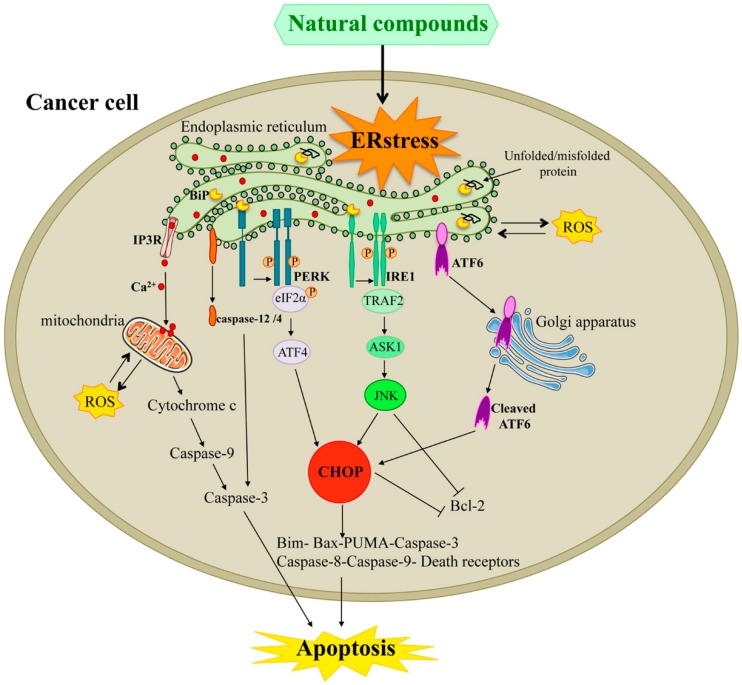
ER stress-related apoptosis triggered by natural compounds. If the adaptive UPR pathway is not able to restore the ER function, upon severe or prolonged ER stress, activation of ER stress sensors can lead to apoptosis. A lot of natural compounds can induce ER stress, which leads to activation of the three ER sensors. Dissociation of BiP from all three sensors PERK, IRE1, and ATF6 leads to generation of their active forms. Active PERK dimerizes, autophosphorylates, and via the eIF2α/ATF4/CHOP pathway, modulates intrinsic and extrinsic apoptosis pathways. Active IRE1 has been demonstrated to induce the expression of Bcl-2 family members both via CHOP and via TRAF2/ASK1/JNK. Cleaved ATF6 can activate the induction of the pro-apoptotic transcription factor CHOP and consequently regulate Bcl-2 family members expression. All three branches of UPR can act concertedly to trigger both mitochondrial and death receptors apoptosis. Moreover, the Ca^2+^ release from ER can activate the ER-resident caspase-12/4, which in its active state, can promote the caspase-3 activation leading ultimately to apoptosis. Moreover, in ER stress conditions, oxidative stress induces the calcium leakage from ER and its subsequent uptake by the mitochondria leading to releasing cytochrome *c* from the mitochondrial matrix. Upon ER stress conditions, Ca^2+^ release from ER and mitochondrial ROS production alter cellular homeostasis and trigger apoptosis. Abbreviations used in Figure 1: ASK1: apoptosis signal-regulating kinase; ATF6: activating transcription factor 6; ATF4: activating transcription factor 4; Bax: (Bcl-2)-associated X protein; Bcl-2: B-cell lynphoma2; BiP: binding immunoglobulin protein; CHOP: C/EBP homologous protein; eIF2α: Eukaryotic initiation factor 2α; ER: endoplasmic reticulum; IP3R: inositol 1,4,5,-triphosphate receptor; TRAF2: tumor necrosis factor receptor-associated factor 2; JNK: JUN N-terminal kinase; ROS: reactive oxygen species.

In this review article, we discuss the ER stress-involving antitumor mechanisms of the most known natural compounds, and of some new natural compounds, in the most common human cancers (Figure 1).

### 4.1. Role of ER Stress in Curcumin-Induced Apoptosis in Cancer

Curcumin is a polyphenol compound extracted from the turmeric rhizome of *Curcuma Longa* plant, and is a yellow spice widely used in Indian cooking, textile dyes, and in traditional Ayurvedic medicine [57]. Over the past ten years, in vitro experiments have demonstrated the anticancer effects of curcumin in different cancer cell lines by inducing cell cycle arrest and apoptosis, most importantly through modulation of several distinct cancer targets [14]. Garrido-Armas et al. recently demonstrated, in A172 human glioblastoma cell line, that curcumin can cause cell death via a paraptosis pathway involving the ER. The authors observed changes in the expression of IRE1α and ATF6 genes, miR27a, miR-222, and miR-449 after exposure of the cells to curcumin [58].

As discussed in different papers, curcumin can induce cancer cell death and the molecular mechanisms of curcumin-induced apoptosis in metastatic prostate cancer cells were recently investigated by Rivera et al. These authors, utilizing a gel-free shotgun quantitative proteomic analysis associated with tandem mass tag isobaric labeling-based-signaling networks, revealed that curcumin promoted ER stress-mediated apoptosis in PC3 prostate cancer cells. The mechanisms by which this compound caused cell death were associated with ROS production, autophagy, and UPR induction, in particular with increased BiP, IRE1α, PDI, and calreticulin expression [59]. Curcumin was also able to induce apoptosis in the WEHI-3 murine myelomonocyte leukemia cell line in a dose-dependent manner. Interestingly, curcumin increased CHOP, ATF6, IRE1, and caspase-12 expression levels. Therefore, curcumin increased ROS production and Ca^2+^ release in the cytosol but decreased the level of mitochondrial membrane potential [60]. Roberts et al., investigating the effects of combined treatment with curcumin and sildenafil in different gastrointestinal tumor cell lines, demonstrated, using siRNA experiments, that PERK and ATF6 are involved in curcumin combined with sildenafil cytotoxicity [61]. In LoVo and HT-29 human colorectal cancer cell lines, curcumin improves the anticancer activity of irinocan by increasing ROS production and by activating the ER stress pathway [62]. The rhizome of many Curcuma species is rich in other phenolic compounds, collectively called curcuminoids, consisting of a mixture of curcumin, demethoxycurcumin (DMC), and bisdemethoxycurcumin (BDMC). In the NCI H460 human lung cancer cell line, BDMC significantly induced apoptotic death as indicated by activation of caspase-3, -8, and -9, and increased ROS levels and Ca^2+^ production, together with increased ER stress associated proteins such as BiP, CHOP, IRE1(-α and -β), ATF6 (-α and -β), and caspase-4 [63]. In the same human lung cancer cell line, DMC was also able to promote apoptosis by activating caspase-3, -8, and -9, and to promote apoptosis-inducing factor (AIF), Endo G and poly (ADP-ribose) polymerase (PARP) expression. Furthermore, its anticancer activity occurs also through GRP78, GADD153, IRE1β, ATF6 (-α and -β), and caspase-4 increased expression [64]. In SW620 colon cancer cells, B63, a mono-carbonyl analogue of curcumin synthetized to increase its biological activity and bioavailability, showed significant anti-proliferative and pro-apoptotic effects by up-regulating the levels of Bad and Bim proteins and enhancing cytochrome *c* release from mitochondria. Moreover, its anticancer activity was dependent on ER stress activation [65]. In two different human ovarian cancer cell lines (A2780 and CP70), the curcumin analogue B19 induced apoptosis that was more effective than curcumin in the activation of caspase-3. At an apoptosis-promoting concentration, B19 induced ROS production and ER stress activation; similar to curcumin, B19 acts through different molecular pathways, including ROS and ER stress [66].

In the H1975 human non-small cell lung cancer (NSCLC) cell line, gefitinib-resistant WZ35, an analog of curcumin, exerts cytotoxic effects by increasing ROS levels and by activating the ER stress pathway [67]. MTH-3, a water soluble curcuminoid derivative, has also been shown to induce intrinsic and extrinsic apoptosis pathways mediated by ER stress signals in the MDA-MB-231 human breast cancer cell line [68].

The activation of ER stress-related apoptosis in cancer cells can open the way to new therapeutic options for curcumin and its analogues in cancer therapy. The ER-stress mediated anticancer activity exerted by curcumin and its analogos is summarized in Table 1.

### 4.2. Role of ER Stress in Resveratrol-Induced Apoptosis in Cancer

Resveratrol, a polyphenolic compound belonging to the class of stilbenes, is present in many plants including grape (mainly skin), blueberries, and peanut, as well as red wine [69]. It has been shown to arrest the cell cycle and to trigger apoptosis by inhibiting ERK1/2 cascade and modulating the expression of some proteins involved in DNA synthesis and the cell cycle, such as p53 and cyclin-dependent kinases [70]. Resveratrol has been found to block cancer growth by targeting different molecules and pathways involved in cancer development [70]. Recent findings demonstrate its ability to induce ER stress-related apoptosis in different cancer cell types. In human multiple myeloma cell lines, resveratrol induces ER stress-related apoptosis by inhibiting the pro-survival XBP1’s function and by promoting the enrichment of its molecular target sirtuin1 [71]. In the A375SM malignant melanoma cell line, resveratrol activates apoptosis and cell cycle arrest through enhancing simultaneously ER stress and ROS production. The A375SM treatment with resveratrol induces the increase of p38, p53, and Bax expression levels and the decrease of Bcl-2 level. Resveratrol also increases the intracellular levels of ER stress-related proteins p-eIF2α and CHOP [72]. In the HepG2 human hepatoblastoma cell line, resveratrol induces ER stress, increasing XBP1 splicing and CHOP expression; moreover, resveratrol intensifies palmitate-induced cell death of HepG2 cells and increases palmitate-induced ER stress. Generally, in this cell line, resveratrol promotes an amplification of palmitate toxicity primarily through ER stress-dependent apoptosis [73]. Moreover, in NCI-H460 human non-small cell lung cancer cell line, Bai et al. demonstrated a pro-apoptotic activity of resveratrol in miR-200c-positive cells. To analyze the mechanism of resveratrol in these cells, by using Target Scan, it was possible to determine the predicted target genes of miR-200c. The authors focused their attention on ER stress-related proteins, such as RECK, a membrane-bound protein, or ER stress molecules, such as BiP and CHOP, which all were observed to be increased in miR-200c-transfected cells, leading to the conclusion that miR-200c expression sensitizes H460cells to resveratrol, mainly through RECK expression [74]. In two human nasopharyngeal carcinoma cell lines, NPC-TW076 and NPC-TW039, resveratrol induced ER stress- and autophagy-related apoptosis as revealed by the marked increase of IRE1, p-PERK, CHOP, and ATF6. Pretreatment of the cells with specific caspase-12 inhibitors but not caspase-4 inhibitors, or silencing caspase-4 or -12 by siRNA for 24 h prior to incubation of resveratrol for 24 h, significantly reduced the activation of caspase-3 and -9 originally induced by resveratrol. In the same study, resveratrol induced autophagy together with UPR expression and ER dilatation [75]. In human lung adenocarcinoma A549 cells, a resveratrol and arsenic trioxide combination promotes ER stress- and mitochondrial dysfunction-mediated apoptosis as manifested by the increased expression of ER stress hallmarks BiP, caspase-12, and CHOP. Moreover, these authors demonstrated that resveratrol and arsenic trioxide-induced ER stress and mitochondrial dysfunction depended on ROS production as indicated by the experiments in the presence of N-acetyl-L-cysteine (NAC), a potent ROS scavenger, that attenuated the expression of ER stress-related markers and the loss of mitochondria membrane potential [76]. Gwak et al. reported that resveratrol prompts ER stress–related apoptosis by interfering with protein glycosylation [77]. However, the use of resveratrol has been limited by its poor solubility and low bioavailability [78]. Drug resistance represents one of the main reasons of the failure of chemotherapy in different cancers, so the development of novel therapeutic strategies is highly mandatory. An effective analogue of resveratrol, (Z)3,4,5,4′-trans-tetramethoxystilbene (TMS), selectively induces cell death of gefitinib-resistant non-small cell lung cancer cells by enhancing the cytosolic [Ca^2+^] levels and causing ER stress (as indicated by PERK and eIF2α activation and CHOP upregulation [79]). A natural demethylated resveratrol analog from blueberries, pterostilbene, exerts anticancer ER stress-related activity in human esophageal cancer. Indeed, pterostilbene upregulates BiP, ATF6, p-PERK, p-eIF2α, and CHOP expression, and increases intracellular calcium levels in EC109 esophageal cancer cells. Silencing CHOP by siRNA significantly decreases the pterostilbene-induced apoptosis. Moreover, in in vivo experiments, this compound was also shown to significantly upregulate BiP and CHOP expression in EC109 xenografts. These experiments provide evidence of ER-mediated pro-apoptotic activity of pterostilbene in human esophageal cancer cells [80]. Res-006, a novel resveratrol derivative, induced cell death in human hepatoblastoma HepG2 cells by triggering ER stress and mitochondrial dysfunction, providing a rationale for the development of new resveratrol-derived molecules for cancer treatment targeting both mitochondria and ER stress [81]. The ER stress-mediated anticancer activity exerted by resveratrol and its analogos is summarized in Table 2.

### 4.3. Role of ER Stress in Green Tea Polyphenols-Induced Apoptosis in Cancer

Green tea extracts (GTE) possess antitumor activity mainly due to the presence of flavan-3-ols, specifically epigallocatechin gallate (EGCG). EGCG decreases cell viability and promotes cell death in many cancer cell lines. While the mechanism of action of GTE has been examined in vitro and in vivo, the molecular targets of green tea catechins remain to be clarified. Findings from the literature indicated that GTE flavan-3-ols target ER function [82].

A GTE extract, Polyphenon E^®^, caused severe and prolonged ER stress in human prostate cancer cells PC3 by activating the PERK signaling arm, as demonstrated by prolonged activation of p-eIF2α and ATF4 [83]. A significant activation of XBP1 mRNA splicing has also been observed in these cells, indicating an involvement of both IRE1α and ATF6 signaling arms of ER stress in the cellular response to Polyphenon E^®^ [84]. XBP1s is generated by an unconventional splicing reaction upon disruption of ER homeostasis and is a marker of ER stress activation contributing to upregulation of CHOP [85]. Polyphenon E^®^ would seem to resensitize cells to chemotherapy compounds, mainly through the increase of the expression of the pro death protein CHOP. This event seems very important in prostate cancer cells, which are more susceptible to ER stress, probably because of the remarkable demand of protein translation and processing, which certainly goes beyond the capacity of cellular folding proteins. [84]. In MM98 malignant mesothelioma cell line, EGCG induces an acute increase of BiP, XBP1, CHOP, and ATF4 with simultaneous activation of caspase-3 and -8 [86]. Among a panel of different ECG and EGCG analogues, JP8 emerged as the most potent autophagy-inducer in B16 melanoma cells. In this cell line, JP8 reduced cell viability and increased apoptosis more effectively than in normal mouse AML-12 hepatocyte cells. Treatment with ATG5 siRNA diminished JP8-induced cell death and in these conditions also stress response proteins such as IRE1α, CHOP, p-eIF2α were downregulated [87].

The ER-stress mediated anticancer activity exerted by green tea polyphenols and its analogos is summarized in Table 3.

### 4.4. Role of ER stress in Tocotrienols-Induced Apoptosis in Cancer

Vitamin E comprises two groups of compounds: α-, β-, γ-, and δ-tocopherols (TPs), and the corresponding unsaturated tocotrienols (TTs). α-Tocopheryl succinate, a derivative of α-tocopherol, induces apoptosis in SGC-7901 human gastric cell line increasing the expression of the main ER stress-associated molecules, BiP, CHOP, and caspase-4. Moreover, in the presence of antioxidant NAC, the mRNA and protein expression of BIP and CHOP was inhibited, indicating that in SGC-7901 cells, α-tocopheryl succinate induced ER stress through ROS production [89]. The other component of vitamin E is represented by TTs. TTs are present in different natural sources such as red palm oil, annatto seeds, and rice bran. Recently, TTs (specifically, γ-TT and δ-TT) have sparked interest due to their health-related activities in chronic pathologies based on their antioxidant, neuroprotective, cholesterol-lowering, and anti-inflammatory roles. Several in vitro and in vivo studies highlighted the antitumor effects of TTs in many types of cancer cells. Especially, TTs were shown to exert anti-proliferative/pro-apoptotic effects, to affect cancer stem cell subpopulation [90], and to decrease the metastatic or angiogenic abilities of different cancer cells [91]. In malignant +SA mammary epithelial cell lines, γ-TT-induced apoptosis is mediated by the PERK/eIF1α/ATF4 ER stress response pathway and by the increased expression of the pro-apoptotic protein CHOP and TRB3 [92]. Another study, focusing on the molecular mechanisms of anticancer activity of γ-TT in breast cancer cell lines, showed that in MDA-MB-231 and MCF-7 cells, γ-TT induced apoptosis, at least in part, mediated by ER stress. In these cells, ER stress induction of JNK and p38 MAPK followed by up-regulation of DR5 in a CHOP-dependent manner was demonstrated to be involved in γ-TT-prompted apoptosis [93]. Patacsil et al. demonstrated that, in the MDA-MB-231 and MCF-7 breast cancer cell lines, γ-TT induced ER stress-mediated apoptosis as demonstrated by gene expression microarray analysis. Indeed, microarray analysis highlighted the modulation of genes involved in the ER stress response, such as ATF3, a target gene for ATF4; the authors demonstrated that ATF3 had a crucial role in γ-TT-induced apoptosis in MCF-7 cells. Moreover, an up-regulation of the ER stress-related protein markers BiP, ATF4, PERK, and IRE1α was observed in both MDA-MB-231 and MCF-7 cells treated with γ-TT [94]. In these cell lines, the cytotoxic activity of γ-TT was associated with the concerted induction of autophagy and ER stress-mediated apoptosis, since γ-TT also activated LC3-I conjugation to ATG5-ATG12 and the conversion of LC3-I in to its lipidated and autophagosome-bound form LC3-II [95].

Comitato et al. demonstrated that, in the HeLa human cervical cancer cell line, and in the MCF-7 human breast cancer cell line, γ- and δ-TT induced apoptosis by triggering signals originating from ER stress. Indeed, after TTs treatment the expression of p-IRE1α, XBP-1, and CHOP was increased. γ- and δ-TT induced the expression of caspase-12 in HeLa cells treated with δ-TT, and this activation was associated with caspase-9 cleavage [96].

The role of δ-TT was investigated by Montagnani Marelli et al. in two different human melanoma cell lines, BLM and A375, using in vitro and in vivo experiments. The authors found that δ-TT exerts a significant anti-proliferative/pro-apoptotic effect on both cell lines but not on normal melanocytes, demonstrating that, in both cell lines, δ-TT induced the expression of different ER stress markers such as BiP PERK, IRE1α, p-EIF2α, ATF4, and CHOP. The cleavage of caspase-4 was also triggered by δ-TT in both melanoma cell lines. In the presence of ER stress inhibitors the pro-apoptotic activity of δ-TT was in part counteracted, underlying the fact that the ER stress pathways could represent a relevant target for melanoma treatment [97]. The ER-stress mediated anticancer activity exerted by tocotrienols is summarized in Table 4.

### 4.5. Role of ER Stress in Garcinia-Induced Apoptosis in Cancer

Mangosteen (*Garcinia mangostana L.*) is a fruit used as a traditional herbal medicine in Southeast Asia for the treatment of inflammation, amenorrhea, and abdominal pain [98], which contains various xanthones including α-mangostin, γ-mangostin, and garcinone E with antitumor effects in different cancer cell lines [99]. Sato et al. primarily hypothesized that the pro-apoptotic activity of α-mangostin was related to the ER stress pathway in PC12 cells. As with thapsigargin, α-mangostin induced apoptosis via sarcoplasmic/endoplasmic Ca^2+^ ATPase (SERCA) inhibition [100]. In the LNCaP and 22RV1 human prostate cancer cell lines, a mangosteen fruit extract (MFE) induced an increased expression of ER stress markers such as PERK, CHOP, IRE1α, BiP, and cleaved caspase-4. At the same time, calnexin, an ER membrane protein guaranteeing accurate protein folding and quality control, was reduced. On the other hand, in normal prostate epithelial cells, MFE exerted an opposite effect by reducing PERK expression, indicating that this extract selectively targets prostate cancer cells. This ER stress induction correlates with increased apoptotic death [101]. In a subsequent paper, the same authors surprisingly observed that after CHOP silencing, α-mangostin-treated 22RV1 cells showed significantly increased cleaved caspase-3 expression. The effect was not observed in LNCaP cells [102]. Another xanthone present in mangosteen fruit, Garcinone E, was able to reduce the proliferation of ovarian cancer cells by triggering ER stress and significantly enhancing the protein expression levels of IRE-1α, XBP-1, BiP, CHOP, and caspase-12. It also exerted anti-migratory and anti-invasion effects, resulting in becoming a potential future candidate as an anti-ovarian cancer compound [103]. Gartanin, a polyphenolic xantone isolated from mangosteen fruit, has been shown to exert antitumor activities in different human malignant cells [104]. Li et al. demonstrated that gartanin activates an apoptotic pathway by modulating the expression of ER stress chaperons and markers in the 22RV1 and LNCaP human prostate cancer cell lines. For the first time, these authors provided evidence suggesting that degradation of androgen receptor (AR) is regulated by the ER stress pathway. CHOP knockdown, indeed, partly reversed the gartanin-induced reduction in AR protein expression, suggesting a possible interplay between UPR activation and AR signaling [105]. Garcinol, a polyisoprenylated benzophenone, derived from *Garcinia indica* exerts antitumor activity in various cancer cells [106]. In Hep3B cells, garcinol induced ROS generation and increased the levels of CHOP; these changes resulted in downstream apoptosis activation with an increase of the Bax/Bcl-2 ratio; a decrease of the mitochondrial membrane potential; the release of cytochrome *c*; and the activation of caspase-9, caspase-3, and their target PARP [106]. Gambogic acid (GA) represents the main active compound of *Garcinia hanburyi*, possessing potent antitumor activity in a broad range of human cancers [107,108]. GA has been shown to induce apoptosis through ER stress activation in the HeLa human cervical carcinoma cell line. GA was able to induce XBP1 splicing and to up-regulate BiP, CHOP, and GADD34 mRNAs. GA induced the ER-related apoptosis pathway through up-regulation of JNK and down-regulation of ERK in HeLa cells [109]. The ER-stress mediated anticancer activity exerted by Garcinia derivates is summarized in Table 5.

### 4.6. Role of ER Stress in Other Natural Compound-Induced Apoptosis in Cancer

Recent papers published in 2018 highlighted the relevance of ER stress in the pro-apoptotic cell death pathway induced by different plan-derived natural compounds in various human cancers.

In human breast cancer cell lines, MCF-7 and MDA-MB 231, pimpinelol, a linear sequiterpene lactone from *Pimpinella haussknechtii*, has been shown to induce apoptosis by increasing protein aggregation and ER stress, as demonstrated by fluorescence microscopy analysis and by mRNA expression of ATF4, CHOP, GADD34, and tribbles-related protein 3 (TRIB3) [110].

Pristimerin is a naturally occurring triterpenoid displaying anti-proliferative effects in different cancer cells [111]. In order to define the mechanisms of action correlated to its antitumor activity, Cevatemre et al. observed an extensive cytoplasmic vacuolation, ER stress induction, and block of autophagic flux in MCF-7 breast cancer cells treated with pristimerin, providing new insights into the mechanisms underlying the activity of pristimerin in breast cancer care [111].

*Cnidium officinale* Makino (COM) has been used as a traditional medicine for thousands of years in Korea, China, and Japan for resolving blood stasis, contusions, and infertility. More recently, COM has been shown to exert antitumor activity in liver, colorectal, and oral cancer. Using Western blot analysis, it has been demonstrated that ER stress-related proteins, such as p-PERK, p-eIF-2α, and ATF4, were modified in apoptotic cancer cells treated with COM. This suggests that ER stress-related proteins play a role in COM-induced cell death. [56].

Although *Salvia miltiorrhiza* (SM) has been reported to have antitumor effects, such as apoptosis induction through caspase activation, cell cycle arrest, anti-angiogenic effect, and Bcl-2 family regulation, the molecular mechanisms of its apoptotic activity need to be demonstrated. Kim et al., in a recent study, showed the activation of ER stress-related apoptosis via miR-216b by the ethanol extract of SM. They suggested that SM induces ER stress by producing ROS and that the activated CHOP expression is followed by an increased miR-216b expression in human multiple myeloma cells (U266 and U937). In addition, SM reduces c-Jun protein expression, which is a target of miR-216b, correlated to the induction of the cleavage of caspase-3 and PARP [112].

Protodioscin (PD) represents the principal steroidal saponin in *Dioscoreae rhizome* exhibiting antitumor effects in several types of human cancer cells [113]. Lin et al., studying the molecular mechanisms associated with PD antitumor activity in human cervical cancer cells, have recently suggested the involvement of mitochondrial dysfunction and BiP/eIF2α/ATF4/CHOP ER stress branch activation. In their study, the authors demonstrated that the silencing of BiP and CHOP by siRNA reversed the augmented ER stress-related protein expression by PD and reduced ER stress-induced apoptosis in cervical cancer cells [114].

Regarding pancreatic cancer cells, by analyzing the anti-cancer effects of *Peonia suffruticosa* (PS) aqueous extract, the researchers found an increased activity of caspase-8, -9, and -3, and inhibition of proteasome activity. They also demonstrated a weak upregulation of death-associated protein kinase 3, an upstream ER stress-responsive integrator of apoptosis and autophagy, suggesting a partial implication of ER stress in PS anticancer activity [115].

The mechanism of the anticancer effects of a leaf methanol extract of *Clinacanthus nutans*, a plant with cytotoxicity against leukemia cells, was evaluated in the human SUP-T1 lymphoma cell line. This extract was able to decrease the mitochondria membrane potential, to induce annexin V overexpression, ROS production, calcium release, and IRE1α and CHOP protein overexpression, indicating that ER stress is one of the pathways involved in the apoptotic cell death of *Clinacanthus nutans* [116].

Chrysophanol, an anthraquinone whose antitumor effects have been shown in many in vitro and in vivo studies [117], has been reported to induce apoptosis in MCF-7 and BT-474 breast cancer cells as indicated by DNA fragmentation. Moreover, in these cell lines, chrysophanol induced ROS production and PERK, eIF2α, IRE1α, and CHOP protein expression. Co-treatment of cells with chrysophanol and NAC reduced the ER stress-related protein expression, indicating that ROS production and ER stress are important pathways in pro-apoptotic chrysophanol activity in breast cancer cells [118]. Many studies analyzed the anticancer activity of garlic and garlic-based extracts. In MDA-MB-231 human breast cancer cells and in WHCO1 human esophageal-cancer cells, the pro-apoptotic activity of ajoene, an allylsulfur compound found in garlic, was associated to its ability to induce an ER accumulation of misfolded proteins and to activate UPR. Using a fluorescently ajoene analogue, the authors demonstrated that this compound targets and accumulates in ER and increases the levels of BiP [119]. Moreover, the same authors demonstrated that in WHCO1 cells, the cytotoxic activity of the ajoene analogue BisPMB depends on increased CHOP expression [120]. Petrovic et al. investigated the molecular mechanism associated with the anticancer activity of ethanol-based garlic extract (GE) in different mammalian cancer cell lines. Using a multiplexed inhibitor bead (MIB) assay, significant changes in GE-treated versus control cells were found for 1000 proteins, and successive KEGG analysis of these proteins indicated that after GE treatment, proteins involved in the ER response were changed. Therefore, in agreement with other studies, GE activates apoptosis in many human cancer cells via ER stress and ROS regulation [121].

The antitumor activity of 7-acetylsinumaximol B (7-AB), isolated from soft coral *Sinularia sandensis*, was evaluated in the NCI-N87 human gastric cell line. 7-AB-treated cells exhibited increased Bad, Bim, Bax, cytochrome *c*, and cleaved caspase-9 and -3 expression levels, suggesting that the apoptotic intrinsic pathway was activated in the 7-AB-induced cell death. Western blot analysis showed increased expression of p-PERK, p-eIF2α, ATF4, CHOP, and p-ATF2 proteins in 7-AB-treated cells. These results indicated that the 7-AB pro-apoptotic activity is partially mediated by the PERK/p-eIF2α/ATF4/CHOP ER stress pathway [122]. 4-nerolidylcatechol, a compound extracted from *Pothomorphe umbellata L.*, has been shown to induce apoptosis via ER stress, both in SK-MEL-28 N and in BRAi/MEKi resistant melanoma cells [123]. Moreover, the role of the monomers PP-22, isolated from *Paris polyphilla*, was investigated on apoptosis and autophagy in nasopharyngeal carcinoma cell line. It has been demonstrated that PP-22 up-regulates PERK, BiP, PDI, ERO1α, IRE, and CHOP proteins, and induces apoptosis via mitochondrial and p38MAPK pathways. Moreover, PP-22 triggered autophagy by inhibiting the ERK signaling pathway in CNE-2 cells [124]. A list of the natural compounds discussed in this section is summarized in Table 6.

## 5. Conclusions

Cancer is one of the prevalent causes of death in the world today. In recent decades, although significant advances have been achieved in cancer therapy, such as chemotherapy, targeted therapy, radiotherapy, surgery, and immunotherapy, these conventional therapeutic approaches are characterized by awful side effects and the development of resistance [125,126,127,128]. Consequently, one of the major purposes for cancer care is to discover innovative therapeutic approaches that are able to selectively destroy malignant cells without damaging normal cells, and to diminish chemotherapy resistance [129,130]. In cancer cells, different conditions, such as hypoxia and lack of glucose, can lead to ER perturbation with an impact on protein folding in the ER, resulting in the accumulation of unfolded proteins, known as ER stress [3,31]. In response to ER stress, cells in the beginning activate an adaptive signaling pathway, called the unfolded protein response, to overwhelm stress and re-establish ER homeostasis [3]. On the other hand, unresolved severe ER stress can lead to the activation of both pathways of apoptosis. Clarifying the mechanisms originated by the different ER stress pathways with the purpose to endorse cell death or cell survival induction represents a significant issue in this field and will support the researchers in the development of new effective drugs for innovative anticancer therapeutic strategies. Natural compounds not only prompt apoptosis but are also able to reduce the resistance to chemotherapy via modulation of the ER stress pathways. Although numerous studies have demonstrated that plant-derived natural compounds exert their anticancer activity by inducing a chronic ER stress, some authors have pointed out that some natural molecules exhibit antitumor activity by inhibiting ER stress-related proteins to reduce adaptative UPR. About this, it has recently been shown that an ethyl acetate extract from *Scindapsus* cf. *hederaceus* (SH-EAE), by decreasing the expression of PERK and IRE1α, exerts anti-proliferative and anti-migratory activities in human lung cancer cells; moreover experiments in zebrafish demonstrated that a decreased expression of PERK and IRE1α in SH-EAE-treated lung cancer cells is accompanied by the reduction of vessels development, suggesting an antiangiogenic effect [131]. The expression of ER stress sensor BiP may be enhanced in metastatic cells, and BiP could represent an advantageous therapeutic target [132]. Although knockdown of BiP by siRNA increases cell death in vitro [133], this may be difficult to reach in vivo and alternative approaches aimed at inhibiting GRP78 may be more effective as therapeutic strategies. In MDA-MB-231 and T-47 human breast cancer cells, EGCG is able to block the ATPase domain of BiP, suppressing its anti-apoptotic function, and sensitizes these cells to etoposide-induced caspase-7 activation and apoptosis [134]. The ER stress-mediated antitumor activity exerted by a great number of natural compounds has been demonstrated in many in vitro studies, as reported in this review, and the cytotoxic effects of these molecules have also been confirmed by in vivo studies. Recently, some studies investigated the relationship between antitumor activity of natural compounds and the ER stress modulation also in in vivo models. Chen et al. found that isoalantolactone, an active sequiterpene naturally present in many vegetables and medicinal plants, induces apoptosis in PC3 and DU145 prostate cancer cells via ROS production and ER stress activation. In vivo, isoalantolactone inhibits DU145 xenograft tumor growth and weight and increases the expression of CHOP in tumor tissue lysates [135]. Betulinic acid, a triterpenoid isolated from *Betula pubescens*, enhances taxol chemosensitivity in breast cancer xenografts; the immunohistochemistry assay revealed that betulinic acid up-regulates the expression of BiP and CHOP, both alone or combined with taxol, confirming the results obtained in vitro [136].

As mentioned, natural compounds provide an important role in anticancer therapy [137,138,139,140,141]. Several epidemiologic studies have highlighted how consumption of plant-based foods, rich in phytochemicals, such as curcumin, resveratrol, and EGCG, are associated with a lower risk of many chronic diseases, including cancer [141,142]. In this review, we presented different studies that suggest how natural compounds can constitute an important arsenal of chemical molecules able to modulate ER stress (both as ER stress inducers and attenuators) to induce cancer cell death. Since the dual role of UPR in tumor progression is well established, it is crucial to understand how and when its modulation can change the balance between pro-survival and pro-death pathways; it has been shown that natural compounds can lead to death both by blocking the adaptive UPR and by promoting sustained ER stress; therefore, since the UPR signaling is a dynamic event, when we examine the ability of natural compounds to modulate ER stress, it is very important to consider the timing and the doses of treatment to be sure to obtain the desired cytotoxic effect.

Nevertheless, further studies are required to better define the molecular mechanisms associated with the anticancer activity of natural compounds in order to convert them to potential effective anticancer drugs.

## Figures and Tables

**Table 1:** Curcumin and its analogues with ER stress-mediated anticancer activity.

Compound	Tumor Type/Cell Line	ER Stress Signaling	Reference
Curcumin (*Curcuma longa*)	Human prostate cancer PC3	↑ IRE1α; BiP; PDI; calreticulin	[59]
Curcumin (*Curcuma longa*)	Murine Myeloma WEHI-3	↑ ATF6; CHOP; IRE1α; caspase-12	[60]
Curcumin + sildenafil (*Curcuma longa*)	Human gastric, colon, liver cancer HC116; HT29; HEPG2	↑ pEIF2α; CHOP	[61]
Curcumin + irinotecan (*Curcuma longa*)	Human colorectal cancer HT29; LoVo	↑ CHOP; PDI; BiP	[62]
Bisdemethoxycurcumin	Human lung cancer NCI H460	↑ BiP; IRE 1(α and β); CHOP; ATF6(α and β); caspase-4	[63]
Demetoxycurcumin	Human lung cancer NCI H460	↑ BiP; IRE 1β; CHOP; ATF6(α and β); caspase-4	[64]
B63 analogue of curcumin	Human colon cancer SW620	↑ ER stress markers	[65]
B19 analogue of curcumin	Human ovarian cancer A2780; CP70	↑ ROS; p-PERK; pEIF2α; CHOP	[66]
WZ35 analogue of curcumin	Human lung cancer HI975	↑ pEIF2α; ATF4; CHOP	[67]
MTH-3 analogue of curcumin	Human breast cancer MDA-MB-231	↑ CHOP; ERO1; PDI; PERK; calnexin ↓BiP	[68]

↑-increasing concentration; ↓-decreasing concentration.

**Table 2:** Resveratrol and its analogues with ER stress-mediated anticancer activity.

Compound	Tumor Type/Cell Line	ER Stress Signaling	Reference
Resveratrol	Human multiple myeloma ANBL-6;	↑ IE1α; CHOP; JNK activation ↓XBP1s	[71]
Resveratrol	Human melanoma A375SM	↑ pEIF2α; CHOP	[72]
Resveratrol + palmitate	Human hepatoblastoma HepG2	↑ ATF4; CHOP	[73]
Resveratrol	Human lung cancer NCI-H460	↑ CHOP; BiP	[74]
Resveratrol	Human nasopharyngeal cancer NPC-TW076; NPC-TW039	↑ IRE 1α; CHOP; ATF6α; p-PERK	[75]
Resveratrol	Human ovarian cancer Pa-1; MDAH2774; SKOV3	↑ PERK;CHOP; IRE 1α; ATF6α; BiP	[77]
Resveratrol + arsenic trioxide	Human lung cancer A549	↑ BiP; CHOP; caspase-12	[76]
RES006 Resveratrol analog	Human hepatoblastoma HepG2	↑ pEIF2α; ATF4; CHOP	[81]
TMS Resveratrol analog	Human lung cancer A579; H1975	↑ pEIF2α; p-PERK CHOP; IRE1α; p-JNK	[79]

↑-increasing concentration; ↓-decreasing concentration.

**Table 3:** Green tea polyphenols and its analogues with ER stress-mediated anticancer activity.

Compound	Tumor Type/Cell Line	ER Stress Signaling	Reference
Polyphenon E^®^	Human prostate cancer PC3	↑ CHOP	[84]
EGCG	Human mesothelioma MM98	↑ BiP; CHOP; ATF4; XBP1	[86]
JP8 EGCG analog	Melanoma B16	↑ ATF4; CHOP	[87]
EGCG	Human bladder carcinoma T24/83	↑ Binding to BiP	[88]

↑-increasing concentration.

**Table 4:** Tocotrienols with ER stress-mediated anticancer activity.

Compound	Tumor Type/Cell Line	ER Stress Signaling	Reference
α-Tocopheryl succinate	Human gastric cancer SGC-7901	↑ BiP; CHOP; caspase-4;	[89]
γ-tocotrienol	Malignant +SA mammary epithelial cell line	↑ p-PERK; p-EIF2α; ATF4; CHOP	[92]
γ-tocotrienol	Human breast cancer MDA-MB-231	↑ BiP; ATF4; CHOP; XBP1	[93]
γ-tocotrienol	Human breast cancer MCF-7; MDA-MB-231	↑ PERK; p-EIF2α; ATF4; CHOP	[94]
γ-tocotrienol	Human breast cancer MCF-7; MDA-MB-231; MCF10A	↑ p-PERK; p-EIF2α; ATF4; CHOP; TRB3	[95]
γ-tocotrienol δ-tocotrienol	Human cervical and breast cancer HeLa; MCF-7	↑ p-IRE1α; XBP1s; CHOP; caspase-12	[96]
δ-tocotrienol	Human melanoma BLM; A375	↑ BiP; CHOP; PERK; IRE1α; p-EIF2α; ATF4; CHOP; caspase-4	[97]

↑-increasing concentration.

**Table 5:** Garcinia derivatives with ER stress-mediated anticancer activity.

Compound	Tumor Type /Cell Line	ER Stress Signalling	Reference
α-Mangosteen	Pheochromocytoma PC12	↓ Ca2+ ATPase activity; ↑ JNK	[100]
Mangosteen fruit extract	Human prostate cancer LNCaP; 22RV1	↑ BiP; PERK; IRE1α calnexin CHOP; caspase-4	[102]
Garcinone-E	Human ovarian cancer HEY; A2780	↑ BiP; IRE1α; XBP1; CHOP; caspase-12	[103]
Gartanin	Human prostate cancer LNCaP; 22RV1	↑ CHOP	[105]
Garcinol	Human hepatocellular carcinoma Hep3B	↑ CHOP	[106]
Gambogic acid	Human cervical carcinoma HeLa	↑ BiP; XBP1s; CHOP; GADD34; JNK	[109]

↑-increasing concentration; ↓-decreasing concentration.

**Table 6:** List of different natural compounds, published in 2018, with ER stress-mediated anticancer activity.

Compound	Tumor Type /Cell Line	ER Stress Signaling	Reference
Pimpinelol (*Pimpinella haussknechtii*)	Human breast cancer MCF-7	↑ ATF4; CHOP; GADD34; TRIB3	[110]
Pristimerin (*Maytenus sp*)	Human breast cancer MCF-7	↑ ATF4; CHOP; IRE1α; pEIF2α	[111]
*Cnidium officinale* Makino	Human myeloid lymphoma U937; U266	↑ p-PERK; pEIF2α; ATF4; CHOP	[56]
*Salvia Miltiorrhiza*	Human myeloid lymphoma U937; U266	↑ p-PERK; pEIF2α, ATF4; CHOP	[112]
Protodioscin *(Dioscoreae rhizome)*	Human cervical cancer HeLa; C33A	↑ BiP; p-PERK; pEIF2α, ATF4; CHOP; JNK	[114]
Paenia suffruticosa	Human pancreatic cancer PANC1; AsPC1; BxPC3	↑ DAPK3	[115]
*Clinacanthus nutans*	Human lymphoma and leukemia SUP-T1; MOLT-4	↑ IRE1α; CHOP	[116]
Chrysophanol	Human breast cancer MCF-7; BT-474	↑ ROS; p-PERK; pEIF2α; CHOP	[118]
Garlic extract	Human multiple myeloma and human prostate cancer RPMI-8226; DU145	↑ BiP; MAPK kinases; RBX1; SKP1	[121]
Ajoene (allyl sulfur compound from garlic)	Human breast cancer and human esophageal cancer MDA-MB-231; WHC1O	↑ BiP; CHOP	[120]
7-Acetylsinumaximol B (*Sinularia sandensis)*	Human gastric cancer NCI-N87	↑ p-PERK; pEIF2α; ATF4; CHOP; p-ATF6	[122]
4-nerolidylcatechol (*Pothomorphe umbellata L*)	Human melanoma SK-MEL-28; BRAi/MEKi SK-MEL-28	↑ p-PERK; IRE1α; BiP; ATF4; CHOP	[123]
PP-22 (*Paris polyphilla*)	Human nasopharyngeal carcinoma CNE-2	↑ PERK; CHOP; BiP; PDI; ERO-LA; IRE-LA	[124]

↑-increasing concentration.

## Abbreviations

7-AB	Acetylsinumaximol B (7-AB)
AR	Androgen receptor
ASK	Apoptosis signal-regulating kinase
ATF6	Activating transcription factor 6
ATF4	Activating transcription factor 4
BiP	Binding immunoglobulin protein
BDMC	Bisdemethoxycurcumin
CaMKII	Calcium/calmodulin-dependent protein kinase II
C/EBP	CCAAT/enhancer binding protein family
CHOP	C/EBP homologous protein
COM	*Cnidium officinale* Makino
DMC	Demethoxycurcumin
GADD34	Growth arrest and DNA-damage-inducible protein 34
GRP78	Glucose-regulated protein 78
eIF2α	Eukaryotic initiation factor 2α
EGCG EMT	Epigallocatechin gallate Epithelial to mesenchimal transition
ER	Endoplasmic reticulum
ERAD	ER-associated degradation
ERO1α	Endoplasmic reticulum oxireductin 1α
ERSE	ER stress response elements
GA	Gambogic acid
GE	Garlic extract
IP3	Inositol 1,4,5,-triphosphate
IP3R	Inositol 1,4,5,-triphosphate receptor
IRE1	Inositol requiring enzyme1
JNK	JUN N-terminal kinase
MFE	Mangosteen fruit extract
PERK	Protein kinase RNA(PKR)-like ER kinase
PDI	Protein disulfide isomerase
ROS	Reactive oxygene species
RIDD	Regulated IRE1-dependent decay
SERCA	Sarcoplasmic/endoplasmic Ca^2+^ ATPase
S1P	Site-1 protease
S2P	Site-2 protease
TPs	Tocopherols
TRAF2	Tumor necrosis factor receptor-associated factor 2
TTs	Tocotrienols
UPR	Unfolded protein response
XBP1	X-box binding protein 1
